# Improving Emergency Department Throughput in a Community Psychiatric Hospital Using a Structured Quality-Improvement Framework

**DOI:** 10.7759/cureus.101254

**Published:** 2026-01-10

**Authors:** Ryan Fudale, Anis Djerdjare

**Affiliations:** 1 Behavioral Health, Bergen New Bridge Medical Center, Thornwood, USA; 2 Clinical Medicine, Filzas Medical Center, New York City, USA

**Keywords:** ed length of stay, emergency department throughput, health systems operations, process improvement, psychiatric emergency care, psychiatric screening, quality improvement

## Abstract

Background

Emergency department (ED) overcrowding and prolonged length of stay (LOS) are pertinent challenges in psychiatric emergency care that have significant implications for patient safety, staff workload, and hospital efficiency. In particular, psychiatric emergency departments experience extended throughput delays due to limited inpatient capacity and external regulatory processes. In New Jersey, the Psychiatric Emergency Screening Services (PESS) is a mandated evaluation process that may contribute substantially to ED LOS, yet its quantitative impact has not been well characterized. This study aimed to quantify the contribution of PESS-related processes to emergency department LOS in a community psychiatric hospital.

Methods

This retrospective observational quality-improvement study analyzed consecutive psychiatric emergency department encounters at Bergen New Bridge Medical Center, a community psychiatric hospital, between April 2022 and March 2023. De-identified operational and electronic medical record data were used to evaluate trends in ED LOS and to assess the contribution of PESS-related processes to overall throughput delays. Quantitative analyses included descriptive statistics and regression-based trend analysis to examine changes in LOS over time and the proportion of LOS that is attributable to PESS intervals.

Results

Across the April 2022-March 2023 study period, emergency department length of stay (LOS) demonstrated substantial variability across encounters. When LOS was aggregated into five-day intervals, linear regression analysis showed a moderate negative association between calendar time and emergency department LOS (r=-0.713, p=1.47 × 10⁻¹²), although notable dispersion persisted throughout the observation period.

Among 118 psychiatric emergency department encounters requiring Psychiatric Emergency Screening Services (PESS) evaluation, the mean emergency department LOS was 22.41 hours (SD 4.90), with a median of 22.42 hours (IQR 19.38-24.94). Mean total PESS evaluation time was 13.38 hours (SD 4.68), accounting for 59.7% of mean total LOS, whereas non-PESS emergency department time accounted for a mean of 9.03 hours (SD 5.85).

Examination of PESS workflow components revealed marked variability within screening-related intervals. Time from PESS order to arrival demonstrated greater dispersion than time from PESS arrival to emergency department departure, contributing substantially to overall variation in total PESS evaluation time.

Conclusion

In this single-center quality-improvement study, externally mandated psychiatric screening processes accounted for a substantial proportion of emergency department length of stay, limiting the effectiveness of hospital-based throughput interventions. These findings suggest that meaningful reductions in psychiatric ED LOS may require system-level policy modifications in addition to internal operational improvements. Quantifying regulatory contributors to throughput delays provides an important framework for future quality-improvement efforts and policy discussions aimed at improving psychiatric emergency care delivery.

## Introduction

Emergency department (ED) overcrowding in the United States has intensified over recent decades, negatively impacting patient safety, clinical outcomes, and hospital efficiency [1]. Increasing patient volumes and hospital admissions have placed sustained strain on emergency services, resulting in prolonged wait times and delayed disposition for many patients [2]. These challenges are particularly pronounced in psychiatric emergency care, where patients frequently experience extended lengths of stay (LOS) compared to those in general medicine EDs [3]. 

Psychiatric emergency departments face many distinct structural and operational constraints that complicate patient flow. Limited availability of inpatient psychiatric beds, heightened safety and monitoring requirements, and complex legal and disposition processes often delay transfer even after clinical stabilization [4]. Psychiatric patients may require continuous observation, specialized staffing, and placement, ultimately reducing flexibility in bed assignment and throughput [5]. As a result, psychiatric EDs are especially vulnerable to overcrowding and prolonged LOS. 

In response to ED overcrowding, hospitals have implemented a variety of internal throughput interventions, including staffing model adjustments, triage redesign, and process standardization initiatives [6]. Prior studies have proven that such strategies can mobilize patient flow under certain institutional conditions, particularly when supported by sufficient staffing and infrastructure [7]. However, the effectiveness of these interventions appears to vary in different hospital settings. In psychiatric hospitals, there are also non-clinical restraints, such as regulatory requirements, that may limit the degree to which hospital-level operational changes alone can meaningfully reduce LOS [8]. 

One underexamined contributor to psychiatric ED throughput delays is the role of state-mandated screening services. In New Jersey, Psychiatric Emergency Screening Services (PESS) are required to evaluate patients for involuntary psychiatric admission and determine appropriate disposition [9]. While PESS plays a critical role in ensuring appropriate and legally compliant psychiatric placement, the timing and availability of these evaluations may introduce substantial delays in ED disposition, particularly when screening resources are limited or demand exceeds capacity [10]. Despite the central role of PESS in psychiatric emergency care, its quantitative contribution to overall ED LOS has not been well characterized. 

Existing studies examining psychiatric ED throughput have largely focused on clinical workflows, staffing models, or inpatient bed availability, with limited attention to the measurable impact of state-mandated screening services [11]. Many analyses aggregate psychiatric and non-psychiatric ED populations, obscuring factors unique to psychiatric emergency care and limiting the applicability of findings to specialized psychiatric settings [12]. There remains a need for institution-specific and data-driven evaluations that isolate system-level contributors to LOS within psychiatric EDs, particularly in community hospitals that function as regional safety-net providers. 

The objective of this study is to quantify system-level contributors to ED LOS in a community psychiatric hospital, with particular emphasis on the contribution of New Jersey's PESS to overall throughput delays. Using a retrospective observational quality-improvement framework, this study evaluates trends in psychiatric ED LOS over time and examines the relative impact of internal operational interventions compared with externally mandated screening services. By identifying dominant drivers of prolonged LOS, this analysis aims to inform future quality-improvement efforts and policy discussions focused on improving psychiatric emergency care delivery.

## Materials and methods

This study was conducted as a retrospective observational quality-improvement analysis at Bergen New Bridge Medical Center (BNBMC), a community psychiatric hospital and safety-net institution located in Paramus, New Jersey, United States. BNBMC operates a psychiatric emergency department serving a large and diverse patient population and functions as a regional referral center for acute psychiatric care. The study evaluated emergency department throughput as part of a hospital-initiated Emergency Department Throughput Project using a Define-Measure-Analyze-Improve-Control (DMAIC) framework.

Study design and population

The analytic cohort consisted of 118 emergency department encounters involving psychiatric presentations that required PESS evaluation during the April 2022-March 2023 study period. All psychiatric emergency department encounters during the study period were eligible for inclusion, and no encounters were excluded. The analysis focused on system-level throughput metrics rather than individual clinical outcomes.

Data sources and collection

Retrospective operational data were generated from institutional hospital records and provided to the study team in de-identified Microsoft Excel (Microsoft, Redmond, Washington) format for analysis. Data included timestamps related to emergency department arrival, clinical processes, and disposition, as well as time intervals associated with PESS. Data were aggregated at the encounter level and reviewed for completeness prior to analysis. Demographic variables were not collected or analyzed as part of this operational quality-improvement study.

Outcome measure

The primary outcome was emergency department LOS, defined as the interval from emergency department arrival to emergency department departure. LOS was measured in hours and calculated for each encounter.

Process metrics and independent variables

To assess contributors to emergency department LOS, multiple throughput-related process metrics were examined. Door-to-triage time was defined as the interval from emergency department arrival to initial triage assessment. Door-to-doctor time was defined as the interval from arrival to the first documented provider evaluation. Door-to-bed time was defined as the interval from arrival to assignment to an emergency department treatment bed. Admission decision-to-departure time was defined as the interval from the clinical decision to admit or transfer to the patient's physical departure from the emergency department. Psychiatric emergency department LOS was defined as the total interval from emergency department arrival to departure.

Psychiatric Emergency Screening Services (PESS)-related time intervals were analyzed separately. Time from PESS order to PESS arrival was defined as the interval from placement of the PESS consultation order to the physical arrival of the PESS clinician in the emergency department. PESS arrival-to-emergency department departure time was defined as the interval from PESS clinician arrival to the patient's departure from the emergency department for inpatient admission, transfer, or discharge. Total PESS-attributable time was defined as the sum of the PESS order-to-arrival and PESS arrival-to-departure intervals.

These variables were selected based on their relevance to psychiatric emergency department workflows and their alignment with the Define-Measure-Analyze-Improve-Control (DMAIC) framework.

Quality improvement framework

DMAIC methodology guided the evaluation process. During the Define phase, operational bottlenecks affecting psychiatric emergency department throughput were identified through multidisciplinary review. In the Measure phase, baseline throughput metrics were established using collected operational data. The Analyze phase involved examining relationships between LOS and key process metrics to identify drivers of delay. In the Improve phase, targeted operational interventions were implemented, including expansion of psychiatric staffing coverage, triage workflow modifications, and communication standardization across emergency department units. The Control phase focused on monitoring throughput metrics over time to assess the sustainability of observed changes.

Statistical analysis

Descriptive statistics were used to summarize emergency department LOS and process metrics, including means and standard deviations. Temporal trends in emergency department LOS across the study period were evaluated using linear regression analysis, with calendar time treated as a continuous variable.

Linear regression analysis was also used to evaluate associations between LOS and key throughput variables, including total PESS time, given the continuous nature of these measures and the interest in estimating the strength and direction of linear relationships. Correlation coefficients were reported, and linear trendlines were fitted to visualize temporal patterns. Statistical significance was defined a priori as a two-tailed p-value of less than 0.05. Confidence intervals were not calculated due to limitations of retrospective aggregate operational data.

The proportion of total LOS attributable to PESS was calculated by dividing the mean total PESS duration by the mean total emergency department LOS. Data management was performed using Microsoft Excel, and statistical analyses were conducted using RStudio (R Foundation, Vienna, Austria).

Ethical considerations

This project was conducted as a quality-improvement initiative using de-identified retrospective operational data. It did not involve interaction with human subjects or use of identifiable private information and was therefore deemed exempt from institutional review board review.

## Results

A total of 118 consecutive psychiatric emergency department encounters were included in the analysis. Emergency department length of stay (LOS) was evaluated across the 12-month study period from April 2022 through March 2023 to assess temporal trends and system-level contributors to prolonged stays.

Emergency department length of stay trends

**Figure 1:**
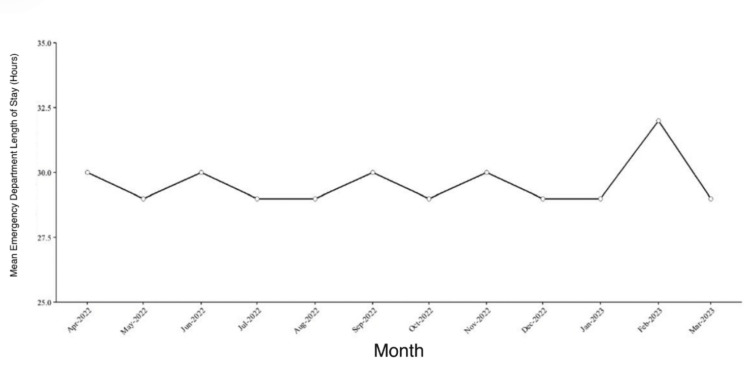
Monthly mean emergency department length of stay during the study period Each point represents the mean emergency department length of stay for a given month.

The mean emergency department length of stay (LOS) over the study period was 22.41 hours (standard deviation (SD) 4.90 hours), calculated at the patient level across all encounters. Monthly mean LOS values fluctuated between approximately 28 and 32 hours and were calculated as unweighted monthly averages. As a result, months with fewer encounters but longer individual stays contribute equally to months with higher encounter volume. Although short-term variation in LOS was observed, no sustained reduction was evident following implementation of hospital-level throughput interventions. The monthly mean emergency department LOS across the study period is shown in Figure 1.

**Figure 2:**
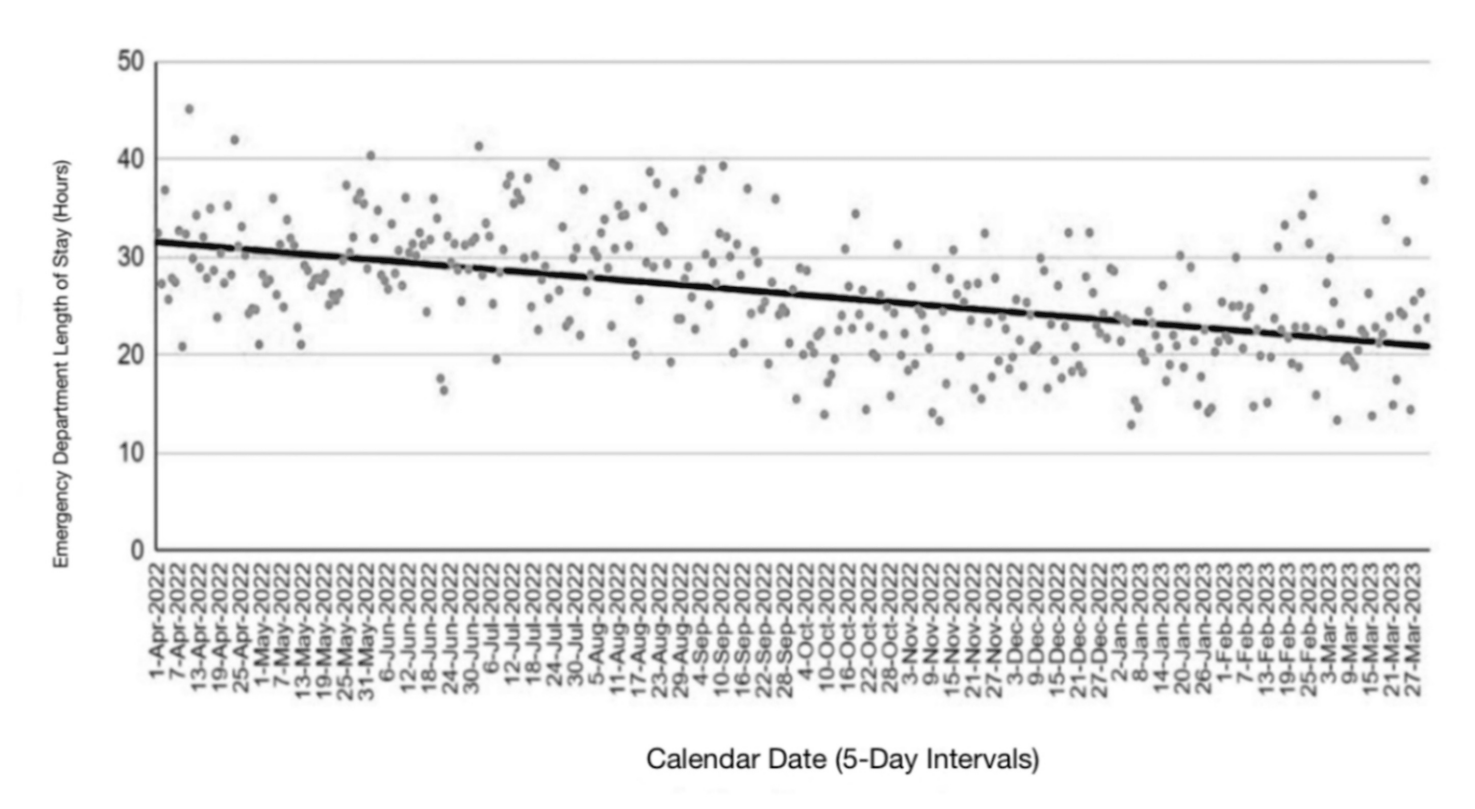
Emergency department length of stay aggregated in five-day intervals Each point represents the mean emergency department length of stay aggregated over five-day intervals across the study period (N=73 intervals). The solid line represents the fitted linear regression trend over calendar time. Linear regression analysis demonstrated a moderate negative association between calendar time and emergency department length of stay (r=-0.713, p=1.47 × 10⁻¹²).

Temporal analysis of emergency department LOS demonstrated substantial day-to-day variability across the study period. When aggregated into five-day intervals, linear regression analysis showed a moderate negative association between calendar time and LOS (r=-0.713, p=1.47 × 10⁻¹²), although considerable dispersion persisted throughout the observation period. Temporal variability in LOS and the associated linear regression trend are illustrated in Figure 2.

Impact of psychiatric emergency screening services

PESS-related processes accounted for a substantial proportion of total emergency department LOS among encounters requiring PESS evaluation.

Among these encounters, overall emergency department LOS demonstrated moderate variability. Mean LOS was 22.41 hours (SD 4.90), with a median of 22.42 hours (IQR 19.38-24.94). When LOS was examined at an aggregate level, total PESS evaluation time comprised a majority of emergency department LOS, whereas non-PESS emergency department time accounted for the remaining portion.

Aggregate summary statistics describing total emergency department LOS and its decomposition into PESS-related and non-PESS time are presented in Table 1.

**Table 1:** Mean emergency department length of stay (LOS) across 118 psychiatric emergency encounters requiring Psychiatric Emergency Screening Services (PESS) evaluation LOS - length of stay; PESS - Psychiatric Emergency Screening Services

Metric	N (encounters)	Mean (hours)	SD	Median (hours)	IQR (hours)	% of total LOS
Total ED LOS	118	22.41	4.90	22.42	19.38–24.94	100%
PESS evaluation time	118	13.38	4.68	13.70	10.37–15.77	59.7%
Non-PESS ED time	118	9.03	5.85	9.51	4.97–13.86	40.3%

Relationship between PESS duration and length of stay

**Figure 3:**
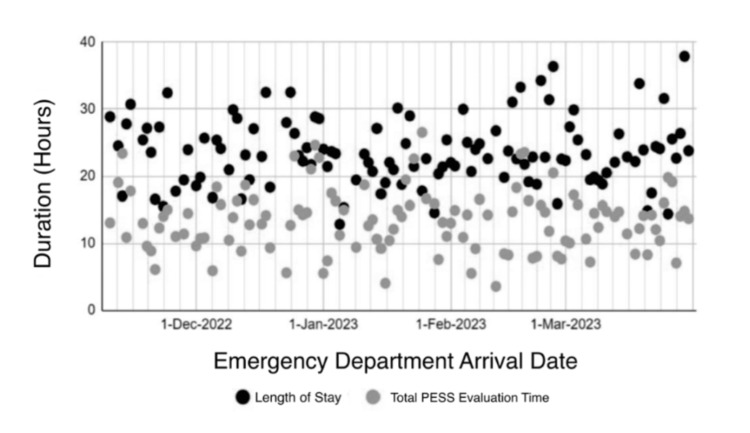
Relationship between emergency department length of stay and total psychiatric Emergency Screening Services (PESS) duration The scatterplot shows individual data points for overall emergency department length of stay (LOS; black) and total Psychiatric Emergency Screening Services (PESS) evaluation time (gray), plotted against patient emergency department arrival dates.

Scatterplot analysis demonstrated a positive association between total PESS evaluation time and overall emergency department LOS, with encounters characterized by longer PESS durations generally exhibiting higher LOS values. The relationship between total PESS evaluation time and emergency department LOS across individual encounters is illustrated in Figure 3.

To further characterize sources of variability within PESS-related time, individual PESS workflow components were examined separately. Time from PESS order to arrival, time from PESS arrival to emergency department departure, and total PESS evaluation time were analyzed to assess their relative distributions and dispersion across encounters. Descriptive statistics for these PESS workflow components are summarized in Table 2.

**Table 2:** Descriptive statistics for emergency department length of stay and Psychiatric Emergency Screening Services (PESS)-related time intervals ED - emergency department; PESS - Psychiatric Emergency Screening Services

PESS workflow component	N (encounters)	Mean (hours)	SD	Median (hours)	IQR (hours)	Min-max (hours)
PESS order to arrival	118	9.45	4.44	9.20	5.90–12.01	1.12–20.70
PESS arrival to ED departure	118	3.93	2.01	3.62	2.78–4.47	0.47–16.81
Total PESS evaluation time	118	13.38	4.68	13.70	10.37–15.77	3.56–26.52

**Figure 4:**
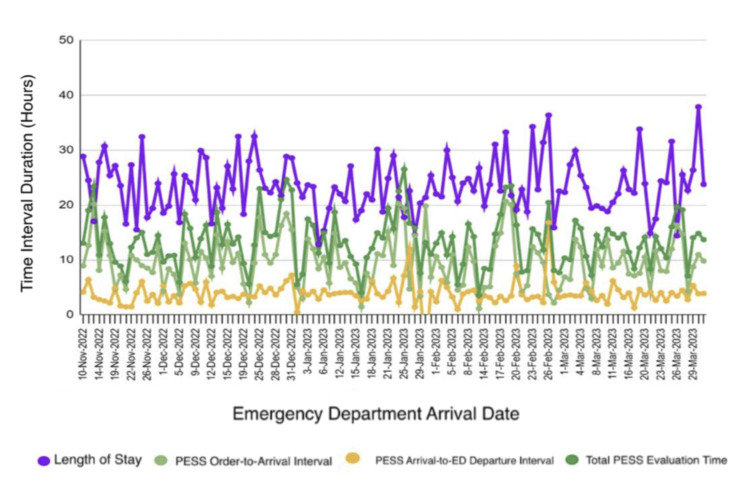
Emergency department length of stay and Psychiatric Emergency Screening Services (PESS) time intervals by arrival date Time series plot showing emergency department length of stay (LOS) and Psychiatric Emergency Screening Services (PESS)-related time intervals for individual encounters plotted by emergency department arrival date. Displayed measures include total LOS, PESS order-to-arrival interval, PESS arrival-to-emergency department departure interval, and total PESS evaluation time, all reported in hours. Across encounters, total LOS ranged from 12.85 to 36.37 hours, PESS order-to-arrival time ranged from 1.12 to 20.70 hours, PESS arrival-to-departure time ranged from 0.47 to 16.81 hours, and total PESS evaluation time ranged from 3.56 to 26.52 hours.

To further characterize variability in these workflow components across individual encounters, temporal patterns in emergency department length of stay and PESS-related time intervals were examined over the study period. Temporal trends in these workflow components are illustrated in Figure 4. 

Summary of key findings

Despite the implementation of multiple hospital-level operational interventions, including expanded psychiatric staffing coverage, triage workflow adjustments, and process standardization, improvements in emergency department LOS were modest and held substantial variability. In contrast, PESS-related processes consistently accounted for a large proportion of total LOS, with delays in screening availability and timing emerging as the dominant contributors to prolonged emergency department stays.

## Discussion

Interpretation of key findings

In this retrospective quality-improvement analysis, externally mandated psychiatric screening processes accounted for a substantial proportion of emergency department length of stay in a community psychiatric hospital. Despite the implementation of multiple hospital-level throughput interventions, overall reductions in LOS were modest and held substantial variability over the study period. In contrast, PESS consistently represented a dominant component of prolonged LOS, accounting for approximately 59.7% of total emergency department time.

Prior studies have consistently shown that psychiatric patients experience longer emergency department lengths of stay than nonpsychiatric populations, largely due to boarding and disposition-related delays [3,4,11]. The present findings are consistent with this literature, demonstrating prolonged LOS despite the implementation of multiple hospital-level throughput interventions. This analysis extends prior work by quantifying the contribution of a state-mandated psychiatric screening process to overall LOS within a community psychiatric hospital setting. Unlike studies that focus primarily on inpatient bed availability or internal emergency department workflows, the present study highlights screening-related processes as a substantial system-level contributor to throughput delays.

The analysis quantifies the relative contribution of screening-related processes to overall throughput but does not imply that PESS is unnecessary or inappropriate. Rather, the results indicate that the timing and availability of mandated screening evaluations are operationally significant and may meaningfully influence emergency department flow in psychiatric settings.

Hospital-level interventions and system constraints

During the study period, several hospital-level operational interventions were implemented as part of ongoing quality-improvement efforts. These included adjustments to psychiatric staffing coverage intended to improve availability during periods of higher demand, modifications to triage workflows to facilitate earlier identification and prioritization of psychiatric patients, and standardization of communication and handoff processes across emergency department and psychiatric services.

Following implementation of these interventions, emergency department LOS demonstrated modest temporal improvement accompanied by substantial variability over time, rather than a consistent or sustained reduction. This pattern suggests that while internal operational changes may influence throughput under certain conditions, their impact may be limited when key components of patient flow depend on externally mandated processes and system-level constraints.

Importantly, this observation does not diminish the value of hospital-based quality-improvement initiatives. Rather, it highlights the practical challenges of achieving durable reductions in psychiatric emergency department LOS in settings where disposition and placement are influenced by regulatory requirements, availability of external screening services, and broader system capacity.

Role of psychiatric emergency screening services in throughput

Psychiatric Emergency Screening Services serve a critical function in ensuring that psychiatric admissions are legally compliant and clinically appropriate [9]. The present analysis does not question the necessity of these services. Rather, it demonstrates that delays occurring prior to screening arrival and variability in screening availability were closely associated with prolonged emergency department LOS.

The observation that screening-related time intervals constituted a large proportion of total LOS underscores the importance of viewing psychiatric emergency throughput as a shared responsibility across multiple system actors. From an operational standpoint, delays related to screening availability may represent a bottleneck that limits the effectiveness of otherwise optimized internal workflows. Quantifying this contribution provides a clearer understanding of where delays occur without attributing fault or intent.

Clinical and operational implications

Prolonged psychiatric emergency department LOS has important implications for patient safety, staff workload, and resource utilization. Extended stays frequently require continuous observation and specialized staffing, which may further constrain emergency department capacity and contribute to crowding. From a clinical perspective, prolonged boarding in emergency settings may delay access to definitive psychiatric treatment and increase patient distress.

The findings of this study suggest that efforts to reduce psychiatric emergency department LOS may benefit from approaches that extend beyond hospital-based workflow optimization to include broader system-level coordination. In settings where screening availability and disposition pathways are governed by external processes, internal operational changes alone may be insufficient to achieve durable improvements. Future investigations that examine alternative screening coordination models, integrate patient-level outcomes, or evaluate throughput across different regulatory environments may help clarify strategies for improving psychiatric emergency care delivery while preserving legal and clinical safeguards.

Limitations and scope of inference

This study has several limitations that should be considered when interpreting the findings. First, the analysis was conducted at a single community psychiatric hospital, which may limit generalizability to other institutions or regulatory environments. Second, the retrospective design and use of aggregate operational data precluded adjustment for patient-level clinical characteristics, acuity, or case mix. Third, the study focused exclusively on throughput metrics and did not assess clinical outcomes or patient experience measures.

Despite these limitations, the study has several strengths. The analysis included consecutive psychiatric emergency department encounters over a 12-month period, reducing the influence of short-term variation. The use of routinely collected, time-stamped operational data enabled objective characterization of emergency department length of stay and screening-related workflow components in a real-world clinical setting.

Additionally, while temporal associations between screening-related delays and LOS were observed, causal relationships cannot be inferred. The results should therefore be interpreted as an operational characterization of throughput patterns rather than an evaluation of screening effectiveness or policy appropriateness.

## Conclusions

In this retrospective quality-improvement study, PESS accounted for a substantial proportion of emergency department length of stay in a community psychiatric hospital, while hospital-level throughput interventions were associated with only modest and variable changes. These findings describe system-level patterns of emergency department flow and should not be interpreted as causal or evaluative of screening effectiveness. Further study is needed to examine how mandated screening processes interact with hospital operations across diverse psychiatric emergency care settings.
